# Lawsuits for Unpaid Medical Bills and the Role of Physician Groups

**DOI:** 10.1001/jamanetworkopen.2025.19763

**Published:** 2025-07-10

**Authors:** Mary Shannon, Kathryn Koch, Molly Metzger

**Affiliations:** 1 Independent researcher, St Louis, Missouri; 2 Independent researcher, Boston Massachusetts; 3 Brown School, Washington University in St Louis, St Louis, Missouri

## Abstract

This cross-sectional study examines the prevalence and impact of physician-driven medical debt lawsuits among Black, low-income, and other marginalized communities.

## Introduction

Medical debt disproportionately affects Black, low-income, and marginalized communities, with far-reaching consequences for financial instability and future health inequities.^1,2^ We examined the prevalence and impact of physician-driven medical debt lawsuits.

## Methods

This cross-sectional study followed applicable STROBE reporting guidelines (eAppendix in Supplement 1). Because our research relied on publicly available legal records, institutional review board approval and informed consent were not required, in accordance with 45 CFR §46.

Using Missouri Case.net,^3^ the state court case-management system, we identified medical debt lawsuits filed from January 2020 through May 2023 by the 5 largest physician groups in the St Louis, Missouri, region (by number of local physicians). Two of the 5 groups appeared in court records (eAppendix in Supplement 1).

In Missouri Case.net,^3^ the label *AC Suit on Account* identifies small-claims cases involving unpaid debts. We classified those cases as medical debt lawsuits. We included only cases filed against individuals, excluding cases unrelated to medical debt (eg, malpractice or breach of contract claims).

We assessed geographic disparities through ordinary least squares (OLS) regression analysis of zip code–level demographics from the 2022 American Community Survey, examining associations between lawsuit volume, total judgment amounts, and wage garnishments (court-ordered paycheck deductions to repay debts). Race and ethnicity data were drawn from the American Community Survey, which classifies demographic characteristics according to self-reported census data, and are included here to examine potential racial disparities in medical debt lawsuits and outcomes. Data were managed using Google Sheets and analyzed with Excel version 1808 (Microsoft) and SPSS statistics version 29 (IBM). Statistical significance was set at 2-sided *P* < .05.

## Results

We identified 973 lawsuits filed by SLUCare Physicians, Washington University Physicians, and their assignees (Table 1). Both operate as units of their parent universities, Saint Louis University and Washington University in St Louis, respectively.

**Table 1.** Medical Debt Lawsuits, Judgments, and Wage Garnishments by Major Physician Groups

Entity	Lawsuits, No. (%)	No. of judgments	Judgment total, $	No. of garnishments	Garnishment total, $
SLUCare Physicians	620 (63.7)	332	717 890	269	589 861
Washington University Physicians	346 (35.6)	198	335 147	157	253 432
Consumer Collection Management^a^	7 (0.7)	1	2356	1	2356
Mercy Clinic	0	0	0	0	0
BJC Medical Group	0	0	0	0	0
SSM Health Medical Group	0	0	0	0	0
Total	973	531	1 055 393	427	845 648

^a^ This organization was an assignee of both SLUCare Physicians and Washington University Physicians.

Of 973 lawsuits, 531 (54.6%) resulted in judgments, of which 462 (87.0%) were default judgments (ie, made without the defendant’s participation). Wage garnishment occurred in 390 default judgments (84.4%) compared with 34 consent judgments (51.5%). Other case dispositions included dismissal (346 cases [36.0%]), court change (34 cases [3.0%]), other (4 cases [0.4%]; tried by court–civil, 3 cases; structured settlement, 1 case), and not disposed or ongoing (61 cases [6.0%]). The mean (SD) judgment was $1987.56 ($2141.63), and the median (IQR) was $1363.42 ($920.23-$2153.76). Majority-Black zip codes, home to 21.9% of the region’s population (286 087 individuals), accounted for 392 identified lawsuits (41.1%), $488 352.10 in judgment totals (47.3%), and 203 wage garnishments (48.0%). Majority-White zip codes, home to 70.8% of the region’s population (924 397 individuals), saw 420 lawsuits (44.0%), $421 515.83 in judgment totals (40.8%), and 160 wage garnishments (37.8%).

OLS regression revealed that majority-Black zip codes had, on average, 17 more lawsuits and an additional $24 314 in judgment compared with majority-White zip codes. Each $1000 increase in median income was associated with a 0.19 decrease in lawsuits and a $242 decrease in judgment (Table 2).

**Table 2.** Factors Associated With Medical Debt Lawsuits, Judgments, and Wage Garnishments Across Zip Codes

Factor	No. of lawsuits, mean (SE) (N = 58)	Total judgments, mean (SE), $ (N = 58)	No. of garnishments, mean (SE) (N = 58)
Model 1			
Majority group^a^			
Black or African American	17.47 (3.35)^b^	24 314 (5039)^b^	10.34 (1.70)^b^
No majority group	17.76 (4.88)^b^	15 502 (7326)^c^	8.17 (2.47)^d^
Population (thousands)	0.59 (0.11)^b^	706 (162)^b^	0.27 (0.06)^b^
*R*^2^	0.47	0.38	0.48
Model 2			
Median income (thousands), $	−0.19 (0.04)^b^	−242 (51)	−0.10 (0.02)^b^
Population (thousands)	0.47 (0.11)^b^	556 (160)^b^	0.20 (0.06)^d^
*R*^2^	0.44	0.36	0.39

^a^ Omitted reference group for majority group is non-Hispanic White.

^b^ *P* < .001.

^c^ *P* < .05.

^d^ *P* < .01.

## Discussion

By underscoring racial and economic disparities in medical debt lawsuits in St Louis, Missouri, with Black and low-income communities bearing the brunt, the findings of this cross-sectional study further illuminate the inequitable impacts of debt collection.^4^ Zip code areas with higher percentages of Black residents and lower median incomes faced significantly more lawsuits, higher judgments, and more wage garnishment orders.

Our analysis raises questions about the alignment of Saint Louis University and Washington University in St Louis, the primary plaintiffs, with community health goals. Both universities report that the suits do not materially impact their financial stability. The same cannot be said for the patients sued.^5,6^ The average judgment was almost $2000, a substantial sum for many families. The higher garnishment rate in default judgments suggests that patients who appear in court may negotiate terms they can afford. However, once entered—by default or consent—judgments allow plaintiffs to pursue garnishment for payment. The findings suggest the need for potential state, local, and federal policy reforms, including requiring improved debt collection transparency and reporting by physician groups, protecting patients from predatory debt collection, broadening health coverage, increasing minimum coverage standards, strengthening medical billing oversight, and increasing financial assistance accessibility.

This study has limitations that should be mentioned. Data in Missouri Case.net may be incomplete or inaccurate, and physician groups may not appear on dockets for cases filed through third-party agencies or under different business names. Also, our zip code–level analysis may obscure individual-level variations. Future research should examine plaintiff experiences, which are notably missing from this analysis.

Despite these limitations, physician groups, especially those affiliated with major academic centers that receive charitable contributions and have community-health obligations, should scrutinize debt collection practices. Through reforms, health care institutions could reduce economic strain and health disparities.

## Notes

Supplement 1. **eAppendix.** Supplemental Methods
eReferences

Supplement 2.
Data Sharing Statement
