# Supplementary material for: Lawsuits for Unpaid Medical Bills and the Role of Physician Groups
Source: JAMA Netw Open. 2025 Jul 10;8(7):e2519763. doi: 10.1001/jamanetworkopen.2025.19763 (PMC12246873; doi:10.1001/jamanetworkopen.2025.19763)
Supplement: Supplement 1. — eAppendix. Supplemental Methods eReferences [file jamanetwopen-e2519763-s001.pdf]

Supplemental Online Content

Shannon M, Koch K, Metzger M. Lawsuits for unpaid medical bills and the role of physician groups. *JAMA Netw Open*. 2025;8(7):e2519763.
doi:10.1001/jamanetworkopen.2025.19763

eAppendix. Supplemental Methods

eReferences

This supplemental material has been provided by the authors to give readers additional information about their work.

© 2025 Shannon M et al. *JAMA Network Open*.

eAppendix. Supplemental Methods

Missouri Case.net Search and Sampling Strategy

We devised a multistep strategy to search Missouri's Case.net portal. Using Boolean operators, we implemented the following approach:

1. *Judicial Circuit, Case Type, and Filing Year Filtering*: We restricted searches to Missouri's 21st and 22nd Judicial Circuits (the circuits for St Louis County and the City of St Louis, respectively), which are home to the litigants of interest to this study, focusing on civil litigation cases filed between January 2020 and May 2023. This timeframe allowed us to analyze medical debt collection lawsuits during the COVID-19 pandemic.
2. *Litigant Name and Alias Variations*:
   a. We conducted multiple search iterations using known fictitious names and aliases of the top-five physician groups in the region (by number of local physicians): Washington University Physicians, Mercy Clinic, SLUCare Physician Group, BJC Medical Group, and SSM Medical Group.[1, 2, 3] Searches included full and shortened of the physician groups (eg, "SLUCare Physicians", "SLUCare") and their affiliated parent institutions (e.g., "Saint Louis University", "SLU").
   b. By activating Case.net's "Include Alias" option, we ensured cases filed under different d/b/a names or by parent institutions were captured. For example, searching "Saint Louis University" with aliases included yielded cases filed by "SLUCare Physicians," while searching for "SLUCare" revealed filings by "Saint Louis University." The following 20 distinct litigant-name variations were utilized: Washington University Physicians, WashU Physicians, Washington University in St Louis, WashU, Mercy Clinic, Mercy Health, Mercy Hospital, SLUCare Physicians, SLUCare, Saint Louis University, SLU, BJC Medical Group, BJC Hospital, Barnes Jewish Hospital, Barnes Jewish, BJC, BJC Health, SSM Health Medical Group, SSM Medical Group, SSM Health.
3. *Inclusion of "AC Suit on Account" Cases*: We reviewed each search result and included cases categorized as "AC Suit on Account," a Missouri court designation typically used for small claims cases involving unpaid debts. In our analysis, this category encompasses medical debt lawsuits filed by physician groups.

© 2025 Shannon M et al. *JAMA Network Open*.

4. *Exclusion of Other Cases*: We excluded cases unrelated to medical debt, such as personal injury/malpractice cases ("CC Pers Injury-Malpractice"), breach of contract cases ("CC Breach of Contract"), wrongful death cases ("CC Wrongful Death"), and other types of cases that were not relevant to the research focus (e.g., "CC Contract-Other," "AC Specific Performance," "CC Other Miscellaneous Actions," "CC Property Damage").

This methodology involved 160 unique queries for the Case.net database. Our approach minimized the risk of overlooking relevant cases while ensuring a targeted focus on the specified judicial circuits and timeframe.

Limitations of Case.net

It is important to acknowledge that, although Missouri Case.net provides a valuable resource for accessing court case information and great care was taken to clean and preprocess data, there still may be instances of incomplete or inaccurately recorded data. Additionally, the system may exhibit biases in the types of cases reported or variations in the level of detail available for different cases.

eReferences

1. Konczal L St. Louis' largest physician groups: Ranked by local physicians. *St Louis Bus J*. March 6, 2020. Accessed December 31, 2024. https://www.bizjournals.com/stlouis/subscriber-only/2020/03/06/st-louis-largest-physician-groups.html

2. Barr D St. Louis' largest physician groups: Ranked by local physicians. *St Louis Bus J*. March 12, 2021. Accessed December 31, 2024. https://www.bizjournals.com/stlouis/subscriber-only/2021/03/12/st-louis-largest-physician-groups.html

3. Simpson M St. Louis' largest physician groups: Ranked by local physicians. *St Louis Bus J*. March 11, 2022. Accessed December 31, 2024. https://www.bizjournals.com/stlouis/subscriber-only/2022/03/11/st-louis-largest-physician-groups.html

© 2025 Shannon M et al. *JAMA Network Open*.
