# Supplementary material for: Lawsuits for Unpaid Medical Bills and the Role of Physician Groups
Source: JAMA Netw Open. 2025 Jul 10;8(7):e2519763. doi: 10.1001/jamanetworkopen.2025.19763 (PMC12246873; doi:10.1001/jamanetworkopen.2025.19763)
Supplement: Supplement 2. — Data Sharing Statement [file jamanetwopen-e2519763-s002.pdf]

Data Sharing Statement

Shannon. Lawsuits for Unpaid Medical Bills and the Role of Physician Groups. *JAMA Netw Open*. Published July 10, 2025. doi:10.1001/jamanetworkopen.2025.19763

Data
Data available: No

Additional Information
Explanation for why data not available: Data originate from Missouri Case.net, a publicly accessible database (https://www.courts.mo.gov/cnet/welcome.do). While we cleaned and structured the data for analysis, the raw data remain publicly available. We also plan to include a more comprehensive analysis in a future publication, which will explore these data in greater depth.
